# Systematic review and network meta-analysis of interventions for adults with simple obesity: Study protocol

**DOI:** 10.1371/journal.pone.0351589

**Published:** 2026-10-08

**Authors:** Lingyao Sun, Lizhen Xu, Dachuang Zhou, Jun Wang, Xuan Yin, Wentao Ye, Lei Tian

**Affiliations:** 1 Centre for Pharmacoeconomics and Outcomes Research, China Pharmaceutical University, Nanjing, China; 2 Department of Endocrinology, Fuzhou University Affiliated Provincial Hospital, Fuzhou, China; 3 Molecular Imaging Center, State Key Laboratory of Drug Research, Shanghai Institute of Materia, Medica, Chinese Academy of Sciences, Shanghai, China; 4 Faculty of Health Sciences, University of Macau, Macau, China; Isfahan University of Medical Sciences, IRAN, ISLAMIC REPUBLIC OF

## Abstract

Obesity has become a major global public health challenge, and its rising prevalence contributes to substantial health and economic burdens. Existing systematic reviews are largely confined to a single intervention category or to mixed populations, which limits targeted recommendations for adults in whom obesity itself, rather than an established metabolic comorbidity, is the principal clinical concern. This protocol describes a systematic review and network meta-analysis comparing dietary, physical activity, behavioural and pharmacological interventions in adults with simple obesity, pragmatically defined as trial populations in which obesity itself is the primary inclusion criterion. We will search PubMed, Embase and the Cochrane Library from inception to the search date for randomised controlled trials. Two reviewers will independently screen records, extract data and assess risk of bias using the Cochrane Risk of Bias 2 tool. The principal primary outcome will be percentage change in body weight at the end of an intervention period of at least 24 weeks. Key secondary outcomes will include the proportion of participants achieving at least 5% weight loss, separate measures of central obesity, and health-related quality of life. Pairwise and network meta-analyses will be conducted under a frequentist random-effects framework, and small-study effects will be examined with funnel plots and regression-based tests. Subgroup analyses and meta-regression will explore prespecified effect modifiers, and the credibility of important subgroup findings will be judged with the ICEMAN instrument. Certainty of evidence will be assessed using the GRADE framework. Depending on the quality, consistency and certainty of the available evidence, the findings may inform clinical decision-making, future guideline development and research priorities. This review is registered with PROSPERO (CRD420251236477).

## Introduction

Obesity has become one of the most important public health challenges worldwide, as documented in multiple reports [1–3]. According to pooled analyses of body mass index trends from 1990 to 2022 by the NCD-RisC collaboration, the burden of adult obesity has continued to rise over the past three decades in most countries. In roughly seventy percent of countries or more, the prevalence of obesity among adults already exceeds that of underweight by a wide margin, and this pattern is equally pronounced in low and middle income countries [1,4]. Obesity is closely associated with a wide range of chronic noncommunicable diseases, including type 2 diabetes, cardiovascular disease, nonalcoholic fatty liver disease, and many forms of cancer, and the combined pattern of diabetes and obesity, often termed diabesity, has been recognised as one of the key drivers of the continuing increase in global disease burden [1,5–7]. If timely and effective interventions are not implemented, obesity and its complications will further increase healthcare expenditure and the broader economic burden, reduce population quality of life and life expectancy, and pose long term challenges to the sustainability of health systems and societies [8,9].Importantly, obesity itself represents a pre-disease state that precedes and predisposes to these cardiometabolic comorbidities. Intervening at this stage, before major complications have developed, offers the greatest potential for prevention and the widest range of available management options, encompassing both lifestyle and pharmacological strategies^5,8^.

In recent years, a substantial body of systematic reviews and network meta-analyses has accumulated on weight management interventions for adults with obesity, but these reviews share two recurring limitations when viewed from the perspective of the pre-comorbidity population described above [10–15]. Most existing reviews are confined to a single intervention category. Within lifestyle interventions, published meta-analyses have separately evaluated aerobic exercise for weight reduction [12,13], and behavioural weight management programmes delivered in primary care settings [12]. Within pharmacotherapy, network meta-analyses and systematic reviews have compared anti-obesity medications by drug class, assessing both efficacy and safety profiles [10,11]. Furthermore, existing reviews that include broader intervention comparisons typically enrol mixed populations in which a substantial proportion of participants have established type 2 diabetes, cardiovascular disease, or other metabolic comorbidities, so that the resulting effect estimates are partly driven by the coexisting conditions and may not be directly applicable to adults with obesity as the primary clinical concern [10,11,15]. These reviews provide valuable within-class comparisons but do not allow clinicians to compare lifestyle against pharmacological options, which is the central decision in early obesity management. As a result, no published network meta-analysis has specifically addressed the pre-comorbidity decision context in adults with simple obesity, where the clinically relevant question is not which drug or which diet performs best within its class, but whether a given patient should begin with a structured lifestyle programme or proceed to early pharmacotherapy. This cross-category comparison requires a network meta-analytic framework and, to our knowledge, has not been undertaken for this population.

Against this background, this study will focus on adults with simple obesity and will conduct a systematic review and network meta-analysis of multiple strategies, including dietary interventions, physical activity interventions, comprehensive lifestyle programmes, and pharmacological treatments. By harmonizing outcome measures and methodological approaches, we aim to compare the weight loss efficacy and safety of different intervention modalities and to explore potential effect modifiers such as comorbidity status, intervention intensity, and duration of follow up, to provide more targeted evidence to inform clinical practice guidelines and health policy decisions.

## Methods

We will conduct a systematic review and network meta-analysis, and the study design is illustrated in Fig 1. This protocol has been registered in PROSPERO (CRD420251236477) and will be reported in accordance with the Preferred Reporting Items for Systematic Reviews and Meta-Analysis Protocols statement [16], as detailed in S6 Checklist.

**Fig 1:**
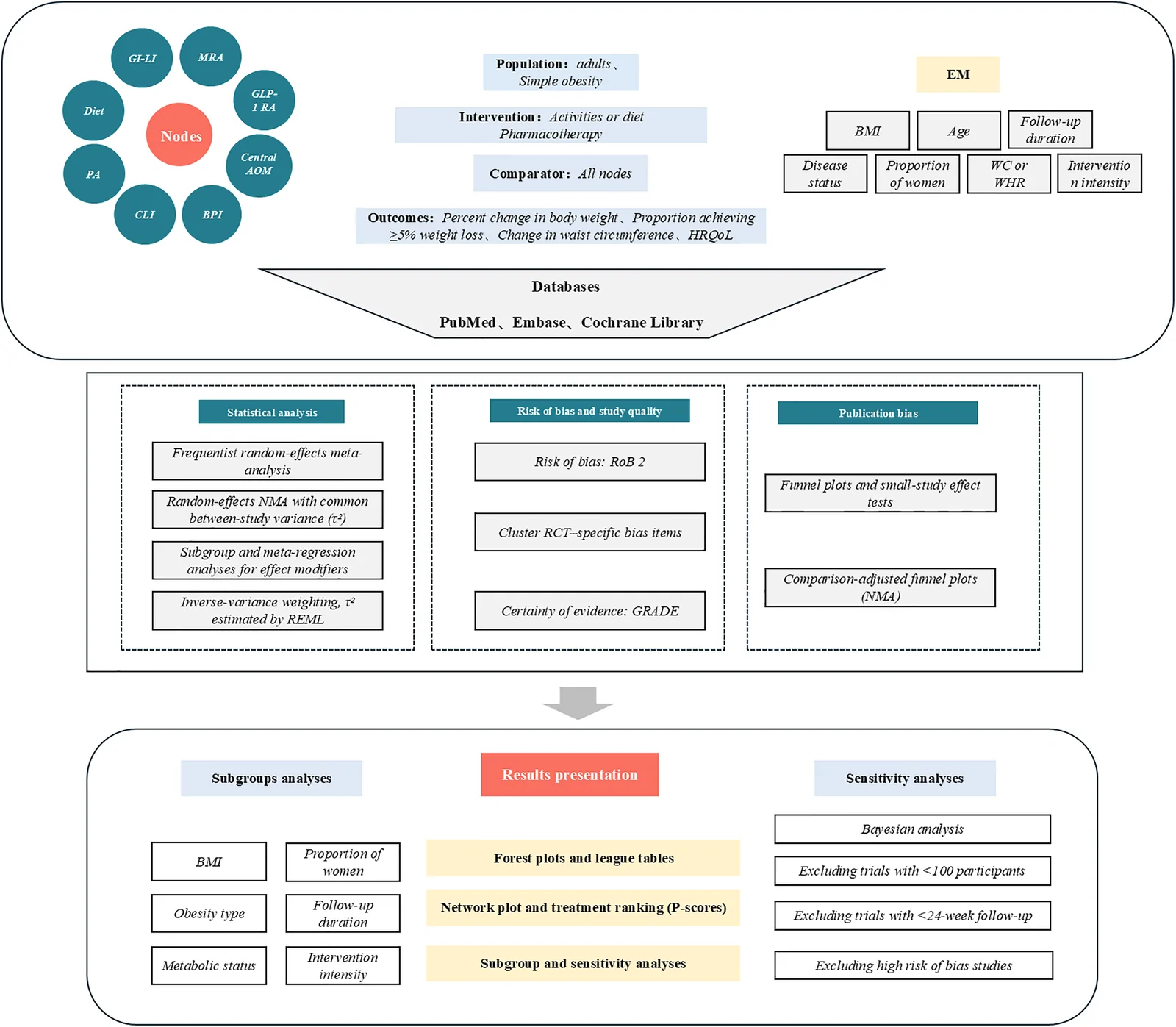
Conceptual study design and analytical framework for the systematic review and network meta-analysis of interventions for adults with simple obesity.

### Eligibility criteria

#### Study design.

We will include parallel group individually Randomized Controlled Trials (RCTs), including cluster RCT. Quasi-randomized trials, cross-over trials, non-randomized controlled studies, case series, cohort studies, case-control designs, animal experiments and in-vitro studies will be excluded.

#### Population.

The target population of this study is adults with simple obesity. Eligible participants will be at least 18 years of age and will meet the diagnostic criteria for adult obesity applicable in the country or region where the trial was conducted. We will extract the obesity definition, BMI eligibility threshold, and baseline mean BMI for each trial. Regional diagnostic criteria and baseline BMI distributions will be examined when assessing transitivity, and baseline BMI will be explored as a potential effect modifier in meta-regression. Obesity must not be attributable to a clearly identified genetic syndrome or endocrine disorder. In addition, trials that use specific chronic conditions such as type 2 diabetes, cardiovascular disease, nonalcoholic fatty liver disease, or malignancy as the primary inclusion criterion will be classified as comorbidity-predominant populations and will not contribute to the primary network. Trials that do not completely exclude these comorbidities but recruit participants from the general community or outpatient settings, and that use obesity itself as the main inclusion criterion without restricting enrolment to a specific disease population, will also be classified as involving adults with simple obesity in order to reflect the characteristics of the general obese population.

We will quantify comorbidity status at the trial level according to the reported proportion of participants with major established comorbidities, principally diagnosed type 2 diabetes and established cardiovascular disease. Trials with a comorbidity proportion below 20% will be classified as predominantly involving adults with simple obesity, trials with a proportion from 20% to <50% as mixed populations, and trials with a proportion of ≥50%, or in which a specific chronic disease is the principal eligibility criterion, as comorbidity-predominant populations. The 20% threshold was selected as a pragmatic boundary informed by epidemiological evidence showing that diagnosed diabetes and established cardiovascular disease generally affect a minority of broadly recruited adults with obesity [17–19], with reported prevalence estimates commonly around or below one fifth, whereas the 50% threshold reflects a majority-based definition of a comorbidity-predominant population. When comorbidity prevalence is not reported, trials in which obesity is the principal eligibility criterion will be classified as predominantly involving adults with simple obesity, given that the background prevalence of major comorbidities in general obesity populations is generally below 20%. All eligible trials will be included in the review irrespective of comorbidity classification, which determines only the analyses to which a trial contributes. The primary network will be restricted to trials with a comorbidity proportion below 20% and to trials in which comorbidity prevalence is not reported and obesity is the principal eligibility criterion. Trials with a comorbidity proportion from 20% to <50%, trials with a proportion of ≥50%, and disease-specific trials will not enter the primary network and will contribute to an all-population sensitivity analysis and to the meta-regression on comorbidity burden as a continuous covariate. A further sensitivity analysis will exclude trials with unreported comorbidity status.

#### Interventions.

Interventions will include all strategies used for weight management that rely on lifestyle measures related to physical activity or diet, behavioural interventions, and pharmacological treatments. Within the network, we will compare the relative effects of all intervention nodes.

Treatment nodes will be defined separately for pharmacological and non-pharmacological interventions. For pharmacological interventions, each eligible individual agent or fixed-dose combination with an explicit indication for weight management will be represented as a separate node rather than being grouped solely according to a broad pharmacological class or mechanism of action. Examples include orlistat [20], liraglutide, semaglutide [21,22], naltrexone/bupropion, phentermine/topiramate [23], and tirzepatide [24]. Different doses of the same pharmacological agent and regimens combining that agent with lifestyle interventions will be assigned to the same drug-level node in the primary analysis. More granular nodes based on pharmacological dose or the addition of an active lifestyle intervention will be created only when the relevant categories are supported by sufficient independent trials and subdivision preserves network connectivity. Fixed-dose combinations will be represented as separate pharmacological nodes and will not be decomposed into their individual components.

For non-pharmacological interventions, we plan four nodes: ① dietary interventions, including energy restriction and structured dietary patterns; ② physical activity or exercise interventions primarily aimed at increasing energy expenditure; ③ behavioural or psychological interventions that promote weight-management-related lifestyle change through cognitive behavioural therapy, motivational interviewing, psychological counselling, or other active behavioural strategies; and ④ multicomponent lifestyle interventions that combine at least two of the following elements: dietary change, physical activity, and behavioural strategies. Assignment to any of these active intervention nodes requires a structured, protocol-driven programme with defined content, frequency, or duration; interventions that do not meet this threshold will not be classified as active non-pharmacological interventions. The primary analyses will use these four non-pharmacological nodes. When prespecified more granular intervention categories are supported by sufficient independent trials and subdivision preserves network connectivity, additional analyses will be conducted using more detailed nodes. Lifestyle features will also be coded as non-exclusive tags and may be examined in sensitivity analyses and meta-regression. These tags will capture dietary content, exercise modality, and active behavioural strategies, while intervention intensity will be classified as low, moderate, or high using intervention-specific criteria based on prescribed intensity, duration, contact frequency, supervision, and component intensity. Detailed non-pharmacological node-adjudication and intensity-classification rules are provided in S2 Appendix and S3 Appendix.

#### Comparators.

Comparators will be classified as active or non-active. Active comparators are pharmacological or non-pharmacological interventions that meet the active-node definitions described above. After excluding such active interventions, non-active comparators will be classified as either placebo or no active intervention. The latter category will include no intervention, usual care, or minimal advice only when these conditions contain no structured dietary, physical activity, or behavioural intervention. Placebo and no active intervention will be combined in the primary analysis to preserve network connectivity and will be analysed as separate comparator nodes in sensitivity analyses when the resulting networks remain sufficiently connected. Combining these two comparator types in the primary analysis assumes that the response observed under placebo, which may be influenced by blinding, expectation, and frequent study contact, is comparable to that observed under no intervention, usual care, or minimal advice; this assumption will be examined in the sensitivity analysis described below.

#### Outcomes.

The principal primary outcome will be percentage change in body weight at the end of the assigned intervention period, which must be at least 24 weeks. The proportion of participants achieving at least 5% weight loss will be considered a key secondary efficacy outcome [25], and absolute change in body weight will be assessed as an additional weight-related outcome. Changes in waist circumference, waist-to-hip ratio, and waist-to-height ratio will be analysed as separate measures of central obesity and will not be treated as interchangeable outcomes. Health-related quality of life will be assessed as a patient-reported secondary outcome [26,27]. Other secondary outcomes will include low-density lipoprotein cholesterol, systolic blood pressure, fasting plasma glucose, serum uric acid, nonalcoholic fatty liver disease indices such as the fatty liver index and controlled attenuation parameter, treatment discontinuation or withdrawal due to adverse events, gastrointestinal adverse events, weight regain, and body image scores [25,28,29]. Using this PICO framework, we aim to systematically evaluate and compare the efficacy and safety of different intervention strategies in adults with simple obesity.

### Search strategy

We will systematically search the following electronic databases: PubMed, Embase via Ovid and the Cochrane Library. Searches will cover the period from database inception to the date on which the search is run. In addition to full journal articles, grey literature sources will also be searched to identify additional or unpublished evidence. These sources will include ClinicalTrials.gov, the International Clinical Trials Registry Platform (ICTRP) Search Portal, doctoral and master’s theses, and conference abstracts. Relevant reference lists of included studies and related systematic reviews and network meta-analyses will also be screened to minimize the risk of missing potentially eligible studies.

The search strategy will combine controlled vocabulary terms and free text terms, using a structured Boolean approach that links four blocks: population, condition, intervention, and study design. Population and condition related terms will include expressions such as “obesity or overweight”, “adiposity”, and “weight loss”. Intervention related terms will cover lifestyle and behavioural interventions such as “diet”, “dietary intervention”, “exercise”, “physical activity”, “behavioural therapy”, and “lifestyle intervention”. They will also include specific drug names and pharmacological classes such as “anti-obesity agents”, “orlistat”, “liraglutide”, “semaglutide”, “tirzepatide”, “phentermine/topiramate”, and “naltrexone/bupropion”. Study design related terms will include “randomized controlled trial”, “clinical trial”, “double-blind”, and “placebo-controlled”. We will apply age limits or age-related terms to restrict the search to adult populations, and no language restrictions will be applied. The complete search strategies and all search terms for each database will be reported in S1 Appendix.

### Study selection

To assess study eligibility, at least two reviewers with training in systematic review methods will independently screen the titles and abstracts of all records retrieved. They will then read the full texts of potentially relevant reports to determine whether the studies meet the inclusion criteria. For each study, the two reviewers will classify the decision as “include,” “exclude” or “uncertain” based on prespecified inclusion and exclusion criteria. Any disagreements will be resolved through discussion. If consensus cannot be reached, a third reviewer will make the final decision. For studies excluded at the full text stage, the specific reason for exclusion will be documented and reported in the final manuscript using a flow diagram and a table.

For studies that report incomplete or missing key information, such as intervention dose, duration of follow-up, outcome data, or baseline covariates, we will contact the original authors to request additional data. If the requested numerical data cannot be obtained, prespecified data-conversion and variance-derivation methods will be applied when the reported statistics permit; studies with insufficient quantitative information after these procedures will be included in the qualitative synthesis only. Detailed conversion and variance-derivation rules are provided in S4 Appendix.

### Data extraction

Data extraction will be performed independently by two reviewers using an electronic spreadsheet based on a standardized data extraction form. Before formal use, we will pilot test the form on at least five eligible randomized controlled trials and refine the items and instructions in light of the pilot results. This process is intended to ensure consistent understanding between reviewers and efficient data collection. The extraction form will draw on the Cochrane data extraction template and will be expanded to address issues specific to adults with simple obesity and to network meta-analysis [30].

From each included study we will extract, at a minimum, the following information: a) Basic study information: name of first author, year of publication, journal or source, country or region, funding source and whether conflicts of interest were reported. b) Study characteristics: study design such as parallel group randomized controlled trial, cluster randomized trial, single central or multicentral design, recruitment and follow up periods, inclusion and exclusion criteria, methods of randomization and masking. c) Participant characteristics: total sample size and numbers in each arm, mean age and standard deviation or range, sex distribution, baseline Body Mass Index (BMI) and obesity category, indicators of central obesity, metabolic status and related information. d) Intervention details: type of intervention, dose or intensity, route of administration, frequency and duration of the intervention, measures of adherence or implementation quality. e) Comparator details: type of control, for example placebo or usual lifestyle advice, specific regimen and duration. f) Primary and secondary outcome measures. g) Information relevant to analysis: any raw data that can be used to calculate effect sizes or variances, including numbers of events, means and standard deviations, medians and interquartile ranges, and related statistics. Where a trial reports more than one analysis population, results based on all randomized participants will be extracted in preference to those restricted to completers.

We will identify multiple reports or duplicate publications arising from the same trial by comparing study characteristics such as author names, study location, trial name, trial identification number and registration number. When multiple reports correspond to a single trial, we will collate information across all reports. The version with the most complete data, the highest methodological quality and the greatest relevance to our review question will be used as the primary data source, while additional reports will be consulted as needed to supplement missing information.

### Risk of bias assessment

We will use the Cochrane RoB 2 recommended by Cochrane to assess the risk of bias at the study level for all randomized controlled trials [31]. At least two reviewers with training in research methods will independently complete the risk of bias assessment and will classify each domain as low risk, some concerns, or high risk. In Domain 3, judgements will take account of the proportion of participants with missing outcome data and of the analysis population and imputation approach reported by the trial, so that trials relying on last observation carried forward or on completer analyses in the presence of substantial attrition are identified as at risk of bias due to missing outcome data. Any disagreements will be resolved through discussion, and if consensus cannot be reached a third reviewer will make the final judgement.

For trials that use cluster randomization, referred to as cluster randomized trials or cluster RCTs, we will extend RoB 2 with additional domains that are specific to this design, with particular attention to the following issues: Recruitment bias, meaning whether all participants were identified before randomization or whether some participants were recruited after the allocation had been revealed. Loss of and imbalance between clusters, including whether the number of randomized clusters was sufficient, whether cluster loss or withdrawal was related to the intervention or outcomes, and whether the analysis appropriately accounted for clustering. Other specific design concerns, such as contamination of the intervention across clusters and co-interventions delivered at the cluster level [32].

### Data synthesis

#### Effect size calculation and assessment of heterogeneity.

Pairwise and network meta-analyses will be conducted under a frequentist random-effects framework. Heterogeneity will be assessed using Cochran’s Q, the between-study variance τ^2^, and I^2^, with a common heterogeneity variance assumed across treatment comparisons and estimated using restricted maximum likelihood [33,34]. Continuous outcomes will be summarized as mean differences when measured on a common scale and as standardized mean differences when different instruments measure the same construct. Binary outcomes will be summarized using risk ratios.

#### Transitivity, consistency, and control of multiplicity.

Transitivity is first supported at the design stage through the node classification framework described above: pharmacological nodes are defined at the level of the individual agent or fixed-dose combination, with different doses of the same agent and regimens combining that agent with lifestyle interventions assigned to the same drug-level node in the primary analysis and subdivided only in additional analyses when supported by sufficient independent trials and when subdivision preserves network connectivity; non-pharmacological interventions require structured, protocol-driven content; and non-active comparators are operationally defined to ensure alignment across intervention categories. We will support the assumptions of transitivity and consistency through both design and analysis. During data preparation we will construct three key summary tables. These will present, by trial, by treatment node, and by actual head to head comparison, the distributions of baseline BMI, waist circumference, age, proportion of female participants, prevalence of metabolic comorbidities, duration of follow up, and risk of bias, all of which are potential effect modifiers. We will then use box plots organised by treatment node and by comparison to visualise the overlap of these variables, complemented by simple analyses such as analysis of variance or appropriate nonparametric tests. This approach will allow us to examine whether different nodes and comparisons generally involve similar types of participants, and in this way to judge whether the transitivity assumption is plausible [35,36].

Network connectivity will be determined by the available direct comparisons. If the overall network is disconnected,for example, because there are limited head-to-head trials linking pharmacological and non-pharmacological nodes, we will analyse each connected component subnetwork separately. We will not present indirect comparisons across disconnected subnetworks; any narrative juxtaposition of results across subnetworks will be interpreted cautiously.

On this basis we will further investigate consistency in the network using both local and global approaches. Locally, we will apply node splitting methods to compare direct and indirect evidence for the same treatment contrast. Globally, we will use design by treatment interaction models to test for inconsistency across the entire network [37].

Regarding multiplicity and inflation of type I error that arise from multiple comparisons, we will not apply formal statistical adjustments. Instead, we will prespecify a limited set of primary outcomes and a small number of key subgroup analyses. All other analyses will be clearly labelled as exploratory. When interpreting the results, we will place greater emphasis on the magnitude and precision of effect estimates and on confidence intervals, and will avoid overinterpreting individual P values [38].

#### Subgroup and meta-regression.

Drawing on previous literature and the clinical questions addressed in this review, we will prespecify the following covariates as potential effect modifiers for subsequent meta regression analyses [39]: ①Baseline BMI, as an indicator of the severity of obesity [25,40]. ②Baseline age, to capture possible differences in response to lifestyle change and pharmacotherapy across age groups [26,41]. ③Duration of follow up or length of the intervention period, in order to explore differences between short term and longer term weight loss effects [42,43]. ④Comorbidity burden, defined as the trial-level proportion of participants with diagnosed type 2 diabetes or established cardiovascular disease, entered as a continuous covariate in meta-regression and, in categorical form, used to distinguish studies primarily involving adults with simple obesity from those with a higher prevalence of comorbidities [25]. ⑤Proportion of female participants, to reflect potential sex related differences in weight loss responses [44,45]. ⑥Indices of central obesity such as waist circumference or waist to hip ratio, to assess whether baseline abdominal obesity modifies intervention effects [26,27]. ⑦Intervention intensity, for example the contact frequency in behavioural weight management programmes, the total number of follow up visits, and the duration of each session, used to capture how different levels of intensity influence the magnitude of weight loss and the maintenance of long term effects [46,47]. Detailed operational definitions and decision rules for intervention intensity classification are provided in S3 Appendix. These covariates will be extracted as far as possible from baseline characteristics reported in the included trials and will be incorporated into a unified meta regression model where data availability permits. For all subgroup findings, we will use the ICEMAN instrument to assess the credibility of subgroup effects, with formal assessment prioritized for the prespecified subgroup analyses of the principal primary outcome, namely those defined by comorbidity burden, intervention duration, and baseline BMI, and for any further subgroup in which a substantial difference in treatment effect is observed [48].

#### Sensitivity analyses.

To test the robustness of our main findings, we will perform several sensitivity analyses. For the principal primary outcome, we will fit Bayesian models and examine whether the conclusions remain stable under plausible prior distributions. We will repeat the primary analysis after including trials with an intervention duration of <24 weeks and after excluding studies judged to be at high risk of bias, and will compare the direction and magnitude of effects with the primary findings. For the principal primary outcome, we will also repeat the analysis with placebo and no active intervention entered as separate comparator nodes whenever the resulting network remains connected, and the results of this analysis will be reported alongside the primary findings. When sufficient studies are available and the relevant networks remain connected, subgroup network meta-analyses will additionally be conducted with trials stratified by intervention duration into 24 to <52 weeks, 52 to <104 weeks, and ≥104 weeks, each trial being assigned to a single stratum according to the duration of its assigned intervention period. If the 24 to <52 weeks stratum contains a sufficient number of trials and yields a connected network, this duration-specific network will be considered as the primary analysis for the principal primary outcome, with the pooled network of all trials of at least 24 weeks retained as a supporting analysis. Intervention duration will also be examined as a potential continuous effect modifier in meta-regression when data permit.

#### Assessment of publication bias.

To assess potential publication bias and small study effects in this review, we will apply both graphical and statistical methods at the level of pairwise comparisons and at the level of the entire network. For pairwise comparisons with a relatively large number of studies, defined as at least ten trials, we will construct conventional funnel plots, using the standard error of the effect estimate on the vertical axis and the effect estimate on the horizontal axis, to visually examine whether the distribution of points is symmetric [49]. At the level of the network meta-analysis, we will draw comparison adjusted funnel plots. This method compares effect estimates across different treatment contrasts and recalibrates them to a common reference, so that studies from all edges of the network can be plotted in a single framework. This allows us to evaluate whether there is evidence of systematic small study effects across the entire network [50].

For statistical testing, and where the number of included studies is sufficient and heterogeneity is not extreme, we will use Egger regression tests or Peters tests to quantify the presence of small study effects. When these tests suggest statistically significant small study effects, we will interpret the findings in combination with the visual appearance of the funnel plots, the methodological quality of the trials, the outcome event rates, and any clinical heterogeneity, rather than attributing all funnel plot asymmetry directly to publication bias [51,52].

#### Certainty of evidence.

After completing the risk-of-bias assessments and the main statistical analyses, we will apply the GRADE approach to rate the certainty of evidence for the principal primary outcome and key secondary outcomes [53]. We will classify the certainty of evidence as high, moderate, low, or very low. In the results and discussion sections, we will clearly report the GRADE rating for each key comparison of the principal primary outcome and key secondary outcomes and will use these ratings when interpreting treatment effects and treatment rankings.

#### Statistical implementation.

Frequentist pairwise and network meta-analyses will be implemented in R using the netmeta package. Multi-arm trials will be analysed while preserving the within-study correlations arising from shared trial arms. For sparse binary outcomes, a continuity correction of 0.5 will be added to all cells of studies with zero events in some but not all arms, whereas studies with zero events in all arms will be excluded from risk-ratio synthesis but retained in descriptive summaries. Treatment rankings will be summarized using P-scores and interpreted alongside effect estimates, 95% confidence intervals, and GRADE certainty assessments rather than as stand-alone evidence of treatment superiority. To support joint interpretation of efficacy and tolerability, mean differences in body-weight change will be plotted descriptively against risk ratios for withdrawal due to adverse events; these plots will not constitute a formal multicriteria decision analysis and will not generate composite benefit–risk scores. Detailed procedures for data conversion and variance derivation and for network meta-analysis are provided in S4 Appendix and S5 Appendix, respectively.

#### Study status and timeline.

This protocol describes the planned methods for an ongoing systematic review and network meta-analysis. Record screening was completed in March 2026, and data extraction began in June 2026 and is expected to be completed in August 2026. Data verification and risk-of-bias assessment are scheduled for October 2026, and generation of the main statistical results is anticipated in December 2026. Subsequent steps will include pairwise meta-analysis, network meta-analysis, subgroup and sensitivity analyses, assessment of publication bias, and GRADE certainty assessment. The present protocol describes the study rationale, eligibility criteria, outcome definitions, intervention classification, and planned analytical methods, but does not report review findings or result-based conclusions. The eligibility criteria, outcomes, intervention-node definitions, and principal statistical methods were prespecified before screening and data extraction began, and any amendments made after PROSPERO registration will be transparently documented.

## Discussion

Over the past year, several protocols for systematic reviews and network meta-analyses have been published that focus on specific weight loss strategies or individual drugs. Examples include an updated network meta-analysis of endoscopic weight loss procedures, a systematic review of electroacupuncture for abdominal obesity, and a systematic review with sequential trial analysis that evaluates adverse events associated with tirzepatide [54–56]. These studies provide in depth syntheses of evidence within their respective intervention domains or pharmacological classes. However, they are usually confined to a single technology or single drug category and rarely offer a systematic comparison of multiple strategies.

Building on the methodological approaches used in these protocols, the present review will focus on adults with simple obesity as the core population and will construct a network meta-analysis framework that spans a wide range of lifestyle and pharmacological interventions. The aim is to provide more comprehensive and comparable evidence to support clinical decision making and health policy. Depending on the consistency and certainty of the available evidence, the findings may inform future research, clinical decision-making, and obesity-management guidance.

## Supporting information

S1 Appendix Search strategies. Full electronic search strategies for PubMed, Embase via Ovid, and the Cochrane Library.
(DOCX)

S2 Appendix Non-pharmacological intervention nodes, adjudication rules, and feature tags. Operational definitions and decision rules for assigning non-pharmacological trial arms to treatment nodes.
(DOCX)

S3 Appendix Intervention intensity classification. Thresholds and rules for classifying intervention intensity as low, moderate, or high.
(DOCX)

S4 Appendix Data conversion and variance derivation. Prespecified rules for converting reported summary statistics, deriving change-from-baseline and percentage-change values, and handling cluster-randomized and multi-arm trials.
(DOCX)

S5 Appendix Statistical methods for the network meta-analysis. Detailed specification of the frequentist random-effects models, transitivity and inconsistency assessment, ranking, and sensitivity analyses.
(DOCX)

S1 Checklist PRISMA-P checklist. (DOCX)
