# Supplementary material for: Systematic review and network meta-analysis of interventions for adults with simple obesity: Study protocol
Source: PLoS One. 2026 Oct 8;21(10):e0351589. doi: 10.1371/journal.pone.0351589 (PMC13648986; doi:10.1371/journal.pone.0351589)
Supplement: Supplementary file 1 [file pone.0351589.s001.docx]

**S1 Appendix. Search strategies**

**PubMed**

| **Concept** | **Search strategy** | **Results** |
| --- | --- | --- |
| #1 | "Obesity"[MeSH Terms] OR "Obesity"[Title/Abstract] OR "Overweight"[MeSH Terms] OR "Overweight"[Title/Abstract] | 497205 |
| #2 | "Obesity"[MeSH Terms] OR "Overweight"[MeSH Terms] | 301487 |
| #3 | "obes*"[Title/Abstract] OR "overweight"[Title/Abstract] OR "adiposity"[Title/Abstract] OR "weight loss"[Title/Abstract] | 570804 |
| #4 | #1 OR #2 OR #3 | 616236 |
| #5 | "Adult"[MeSH Terms] | 8597337 |
| #6 | "Randomized Controlled Trial"[Publication Type] OR "Clinical Trial"[Publication Type] OR "Double-Blind Method"[MeSH Terms] OR "Single-Blind Method"[MeSH Terms] OR "randomized"[Title/Abstract] OR "randomly"[Title/Abstract] OR "Clinical | 1866291 |
| #7 | "Anti-Obesity Agents"[MeSH Terms] OR "obesity/drug therapy"[MeSH Terms] OR "antiobesity"[Title/Abstract] OR "anti-obesity"[Title/Abstract] OR "orlistat"[Title/Abstract] OR "lorcaserin"[Title/Abstract] OR "phentermine"[Title/Abstract] OR "topiramate"[Title/Abstract] OR "bupropion"[Title/Abstract] OR "naltrexone"[Title/Abstract] OR "liraglutide"[Title/Abstract] OR "semaglutide"[Title/Abstract] OR "exenatide"[Title/Abstract] OR "dulaglutide"[Title/Abstract] OR "lixisenatide"[Title/Abstract] OR "metformin"[Title/Abstract] OR "canagliflozin"[Title/Abstract] OR "dapagliflozin"[Title/Abstract] OR "empagliflozin"[Title/Abstract] | 95089 |
| #8 | "diet reducing"[MeSH Terms] OR "diet mediterranean"[MeSH Terms] OR "Caloric Restriction"[MeSH Terms] OR "diet*"[Title/Abstract] OR "calorie restriction"[Title/Abstract] OR "energy restriction"[Title/Abstract] | 799134 |
| #9 | "Motor Activity"[MeSH Terms] OR "Physical Fitness"[MeSH Terms] OR "physical activity"[Title/Abstract] OR "exercise"[Title/Abstract] OR "training program*"[Title/Abstract] OR "exercise program*"[Title/Abstract] | 761884 |
| #10 | "Behavior Therapy"[MeSH Terms] OR "Health Education"[MeSH Terms] OR "Health Promotion"[MeSH Terms] OR "behavioral intervention*"[Title/Abstract] OR "lifestyle intervention*"[Title/Abstract] | 389139 |
| #11 | #7 OR #8 OR #9 OR #10 | 1897327 |
| #12 | #4 AND #5 AND #6 AND #11 | 18805 |

Embase (via Ovid)

| **Concept** | **Search strategy** | **Results** |
| --- | --- | --- |
| #1 | 'obesity'/exp OR 'obesity' OR 'overweight'/exp OR 'overweight' | 969665 |
| #2 | 'obesity'/exp OR 'overweight'/exp | 810443 |
| #3 | obes*:ti,ab OR overweight:ti,ab OR adiposity:ti,ab OR 'weight loss':ti,ab | 869672 |
| #4 | #1 OR #2 OR #3 | 1140142 |
| #5 | 'adult'/exp | 13291148 |
| #6 | 'randomized controlled trial'/exp OR 'clinical trial'/exp OR 'double blind procedure'/exp OR 'single blind procedure'/exp OR randomized:ti,ab OR randomly:ti,ab OR 'clinical trial':ti,ab OR 'controlled trial':ti,ab OR 'randomized controlled trial':ti,ab | 3640781 |
| #7 | 'antiobesity agent'/exp OR 'obesity drug therapy' OR antiobesity:ti,ab OR 'anti-obesity':ti,ab OR orlistat:ti,ab OR lorcaserin:ti,ab OR phentermine:ti,ab OR topiramate:ti,ab OR bupropion:ti,ab OR naltrexone:ti,ab OR liraglutide:ti,ab OR semaglutide:ti,ab OR exenatide:ti,ab OR dulaglutide:ti,ab OR lixisenatide:ti,ab OR metformin:ti,ab OR canagliflozin:ti,ab OR dapagliflozin:ti,ab OR empagliflozin:ti,ab | 151751 |
| #8 | 'reducing diet'/exp OR 'mediterranean diet'/exp OR 'caloric restriction'/exp OR diet*:ti,ab OR 'calorie restriction':ti,ab OR 'energy restriction':ti,ab | 1044096 |
| #9 | 'physical activity'/exp OR 'physical fitness'/exp OR 'physical activity':ti,ab OR exercise:ti,ab OR 'training program*':ti,ab OR 'exercise program*':ti,ab | 1218882 |
| #10 | 'behavior therapy'/exp OR 'cognitive therapy'/exp OR 'health education'/exp OR 'health promotion'/exp OR 'behavioral intervention*':ti,ab OR 'lifestyle intervention*':ti,ab | 593498 |
| #11 | #7 OR #8 OR #9 OR #10 | 2771969 |
| #12 | #4 AND #5 AND #6 AND #11 | 43,840 |

**Cochrane Library (CENTRAL)**

| **Concept** | **Search strategy** | **Results** |
| --- | --- | --- |
| #1 | (obes* OR overweight OR adiposity OR "weight loss"):ti,ab,kw | 78524 |
| #2 | 'obesity'/exp OR 'overweight'/exp | 810443 |
| #3 | MeSH descriptor: [Adult] explode all trees | 636816 |
| #4 | #1AND #2 AND #3 | 21736 |
| #5 | randomized:ti,ab,kwOR randomly:ti,ab,kwOR "clinical trial" .ti,ab,kwOR "controlled trial".ti.ab.kwOR "randomized controlled trial":ti.ab,kw | 1468479 |
| #6 | #4 AND #5 | 17551 |
| #7 | MeSH descriptor: [Anti-Obesity Agents] explode all trees | 1084 |
| #8 | "obesity drug therapy".ti, ab,kw OR antiobesity:ti,ab,kw OR "anti-obesity".ti,ab,kw OR orlistat:tiab,kw OR lorcaserin.ti.ab.kw OR phentermine.ti.ab.kw OR topiramate:ti,ab,kw OR bupropion:ti,ab,kw OR naltrexone:ti,ab,kw OR liraglutide:ti,ab,kw OR semaglutide:ti,ab,kw OR exenatide:ti,ab,kw OR dulaglutide: ti,ab,kw OR lixisenatide.ti,ab,kw OR metformin:ti,ab,kw OR canagliflozin:ti,ab,kw OR dapagliflozin:ti,ab,kw OR empagliflozin:ti,ab,kw | 32000 |
| #9 | #7 or#8 | 32233 |
| #10 | MeSH descriptor. [Diet, Reducing] explode all trees | 2455 |
| #11 | MeSH descriptor: [Diet, Mediterranean] explode all trees | 1010 |
| #12 | MeSH descriptor: [Caloric Restriction] explode all trees | 1241 |
| #13 | diet*:ti.ab.kw OR "calorie restriction".ti,ab,kw | 125016 |
| #14 | #10 or #11 0r#12 OR #13 | 125117 |
| #13 | diet*.ti,ab,kw OR "calorie restriction".ti,ab,kw OR "energy restriction":ti,ab,kw | 125016 |
| #14 | #10 or #11 0r#12 OR #13 | 125117 |
| #15 | MeSH descriptor: [Motor Activity] explode all trees | 45044 |
| #16 | MeSH descriptor: [Physical Fitness] explode all trees | 5172 |
| #17 | "physical activity":ti,ab,kw OR exercise:ti,ab,kw OR "training program*".ti,ab,kw OR "exercise program*" ti,ab,kw | 182123 |
| #18 | #15 or #16 or #17 | 189249 |
| #19 | MeSH descriptor: [Behavior Therapy] explode all trees | 26457 |
| #20 | MeSH descriptor. [Cognitive Behavioral Therapy] explode all trees | 15113 |
| #21 | "lifestyle intervention*".ti,ab,kw | 5820 |
| #22 | "behavioral intervention*".ti.ab.kw OR "lifestyle intervention*".ti,ab,kw | 10869 |
| #23 | #22 or #18 or #14 OR #9 | 334739 |
| #24 | #6 AND #23 | 11933 |
