# Supplementary material for: Systematic review and network meta-analysis of interventions for adults with simple obesity: Study protocol
Source: PLoS One. 2026 Oct 8;21(10):e0351589. doi: 10.1371/journal.pone.0351589 (PMC13648986; doi:10.1371/journal.pone.0351589)
Supplement: Supplementary file 2 [file pone.0351589.s002.docx]

**S2 Appendix. Non-pharmacological intervention nodes, adjudication, and feature tags**

Active non-pharmacological interventions will be mapped into four prespecified nodes:

1. Dietary interventions
2. Physical activity or exercise interventions
3. Behavioral or psychological interventions
4. Multicomponent lifestyle interventions that combine at least two structured elements among dietary change, physical activity, and behavioral strategies.

To improve reproducibility and support subsequent sensitivity analyses, we will apply a two-step approach.

Step 1. Node assignment, mutually exclusive

Each trial arm will be assigned to exactly one node using the following decision rules.

1. Identify whether each of the following structured elements is present in the arm:

Diet element present, if either the Energy restriction tag, ER, or the Dietary pattern tag, DP, is assigned as 1, or if another clearly structured diet prescription is reported beyond generic advice.

Physical activity element present, if any of Aerobic, Resistance, or Lifestyle PA is assigned as 1.

Behavioral element present, if any active behavioral strategy is assigned as 1, including Planning, Monitoring Feedback, Coaching Counselling, or Incentives. Education alone without any active behavioral strategy will not be counted as a behavioral element for node assignment.

2. Assign the node as follows:

Multicomponent lifestyle nodes, if at least two structured elements are present.

Otherwise, assign the node based on the single structured element present: Dietary node if only the diet element is present; Physical activity or exercise node if only the physical activity element is present; behavioral or psychological node if only the behavioral element is present.

Arms that reflect usual care or minimal advice will be classified as comparators according to the protocol definitions and will not be assigned to the four active non-pharmacological nodes.

Step 2. Feature tagging, non-exclusive

Within each assigned node, prespecified features will be coded using binary tags for subsequent sensitivity and subgroup analyses. Tags can co-occur within an arm.

2.1 Dietary intervention: adjudication rules and tags

2.1.1 Energy restriction tag, ER

Definition: ER captures dietary interventions whose core mechanism is an explicit calorie deficit.

Assign ER = 1 if the intervention includes any of the following, with any single criterion sufficient:

- A stated daily energy target
- A stated energy deficit
- A very low-calorie diet or another explicit low-calorie prescription
- Meal replacement or portion-control tools explicitly intended to create a calorie deficit, even if kcal values are not stated

2.1.2 Dietary pattern tag, DP

Definition: DP captures dietary interventions defined by a reproducible dietary pattern or a structured food-group prescription.

Assign DP = 1 if the intervention includes any of the following, with any single criterion sufficient:

- A named dietary pattern
- A reproducible food-group prescription or structured food list with explicit emphasis and restriction rules
- Another clearly specified pattern dose or intensity, such as quantified servings, scoring targets, or structured menus

2.1.3 Dual-tagging and diet subtypes for sensitivity analyses

Because many dietary trials combine energy restriction with a structured pattern, each dietary arm will be dual tagged using ER and DP.

If data permits, dietary arms may be subdivided for sensitivity analyses into:

- ER-only, ER = 1 and DP = 0
- DP-only, ER = 0 and DP = 1
- ER plus DP, ER = 1 and DP = 1

Exclusion rule for DP integrity: interventions that primarily differ by single supplements or functional products without a structured diet prescription will not be considered DP and will be handled according to the protocol eligibility criteria.

2.2 Physical activity or exercise intervention: adjudication rules and tags

2.2.1 Aerobic exercise tag, Aerobic

Assign Aerobic = 1 if the arm includes endurance-type activities and reports at least one dose parameter, such as frequency, duration, or intensity.

2.2.2 Resistance training tag, Resistance

Assign Resistance = 1 if the arm includes resistance or strength training, or provides structured prescription details or progression.

2.2.3 Lifestyle physical activity tag, Lifestyle PA

Assign Lifestyle PA = 1 if the intervention aims to increase daily activity or reduce sedentary time and includes quantified targets or tool support.

Multi-tagging rule: physical activity arms may be tagged with more than one subtype.

2.3 Behavioral or psychological intervention: adjudication rules and BCT-module tags

Behavioral components will be described using behavior change technique language and coded using module-level tags. Tags can co-occur.

2.3.1 Planning and goal setting module tag, Planning

Assign Planning = 1 if the intervention includes goal setting, action planning, implementation intentions, problem solving, relapse prevention, or structured coping planning.

2.3.2 Monitoring and support module tags

- Monitoring Feedback: Assign Monitoring Feedback = 1 if participants are required to record weight, diet, or activity and receive feedback from a coach, system, or group.
- Coaching Counselling: Assign Coaching Counselling = 1 if there are regular follow-ups that include counselling or coaching, such as motivational interviewing, CBT-oriented strategies, or case management.

2.3.3 Incentives and reinforcement module tag, Incentives

Assign Incentives = 1 if rewards, points, cash, or non-cash incentives are explicitly tied to goals, such as steps, weight change, attendance, or adherence targets.

2.3.4 Education or information provision intensity tags

Node assignment note: Education is coded as a feature tag only. Education alone will not be counted as a behavioral element for node assignment. For node assignment, behavioral elements are defined by active behavioral strategies, including Planning, Monitoring Feedback, Coaching Counselling, and Incentives.

Education: Assign Education = 1 if educational content is delivered, such as lectures, handouts, printed or digital materials, online courses, or structured informational sessions.

Low Intensity Education: Assign Low Intensity Education = 1 if Education = 1 and no active behavioral strategy is present, namely Planning = 0, Monitoring Feedback = 0, Coaching Counselling = 0, and Incentives = 0. This tag indicates education-only delivery without active behavioral components.

Behavioral Counselling Programme: Assign Behavioral Counselling Programme = 1 if Education = 1 and at least one active behavioral strategy is present, namely any of Planning, Monitoring Feedback, Coaching Counselling, or Incentives is 1. Retain the specific module tags in addition to this programme-level tag.

Multi-tagging rule: behavioral or psychological intervention arms may be tagged with more than one module.
