# Supplementary material for: Systematic review and network meta-analysis of interventions for adults with simple obesity: Study protocol
Source: PLoS One. 2026 Oct 8;21(10):e0351589. doi: 10.1371/journal.pone.0351589 (PMC13648986; doi:10.1371/journal.pone.0351589)
Supplement: Supplementary file 3 [file pone.0351589.s003.docx]

**S3 Appendix. Intervention intensity classification**

To support pre-specified effect modification analyses and meta-regression, each intervention arm will be classified into one of three intensity levels: High, Moderate, or Low. Intensity classification will be applied only after an intervention has met the criteria for assignment to an active non-pharmacological intervention node. This appendix provides operationalized definitions and decision rules for intensity classification across intervention types.

General principles

Single primary indicator: Each intervention type uses one or two core indicators as the primary basis for classification, selected for high reporting frequency and clinical relevance.

Three-level classification: High, Moderate, and Low intensity, with pre-specified thresholds based on existing evidence and guidelines.

Hierarchy rule: When multiple indicators are available and conflict, the higher intensity level is assigned.

Missing data rule: If the primary indicator is not reported and alternative indicators are insufficient for classification, the arm is coded as Intensity = Cannot determine and excluded from intensity-stratified analyses.

3.1 Dietary intervention intensity

Primary indicators: contact frequency and energy restriction level.

3.1.1 Classification criteria

High intensity: Assign Intensity = High if any of the following criteria are met: individualized dietary consultation or contact at least once per week; very low-calorie diet (VLCD, ≤800 kcal/day) or total meal replacement program; or prescribed energy deficit of at least 750 kcal/day.

Moderate intensity: Assign Intensity = Moderate if any of the following criteria are met and no High criteria are met: consultation or contact 1 to 3 times per month (less than once per week); prescribed energy deficit of 500 to 749 kcal/day; or structured meal plan with regular monitoring.

Low intensity: Assign Intensity = Low if any of the following criteria are met and no High or Moderate criteria are met: contact less than once per month or baseline session only; prescribed energy deficit less than 500 kcal/day or no explicit energy target; or written materials only or general dietary advice without structured follow-up.

3.1.2 Special rules

VLCD or total meal replacement programs are automatically classified as High intensity regardless of contact frequency.

When contact frequency and energy restriction level suggest different intensity levels, assign the higher level.

3.2 Physical activity or exercise intervention intensity

Primary indicator: total weekly exercise duration (minutes per week).

3.2.1 Classification criteria

High intensity: Assign Intensity = High if the intervention prescribes at least 200 minutes per week. When duration is not reported, High intensity may be assigned if the intervention includes frequency of at least 5 sessions per week with duration of at least 40 minutes per session, or supervised training at least 3 sessions per week. This threshold is consistent with the upper range of evidence-based recommendations for weight management.

Moderate intensity: Assign Intensity = Moderate if the intervention prescribes 100 to 199 minutes per week. When duration is not reported, Moderate intensity may be assigned if the intervention includes frequency of 3 to 4 sessions per week with duration of 20 to 39 minutes per session, or supervised training 1 to 2 sessions per week. This range corresponds to standard physical activity guidelines for general health benefits.

Low intensity: Assign Intensity = Low if the intervention prescribes less than 100 minutes per week. When duration is not reported, Low intensity may be assigned if the intervention includes frequency of less than 3 sessions per week or duration of less than 20 minutes per session, or advice to increase activity without specific prescription. This level represents subthreshold exercise doses.

3.2.2 Special rules

For combined aerobic and resistance programs, sum the durations of both components before classification.

For lifestyle physical activity interventions using step goals: assign Moderate if the target is at least 10,000 steps/day; assign Low if the target is less than 10,000 steps/day or if no specific target is provided.

Supervised training may be upgraded by one level relative to unsupervised training of equivalent duration, at extractor discretion with documentation.

3.3 Behavioral or psychological intervention intensity

Primary indicators: total number of contact sessions and total contact time.

3.3.1 Classification criteria

High intensity: Assign Intensity = High if the intervention includes at least 14 sessions or at least 420 minutes (7 hours) of total contact time. Typical characteristics include weekly or biweekly contact, at least 3 behavior change technique (BCT) modules, and combined individual plus group format. The threshold of 14 or more sessions is consistent with the definition of high-intensity behavioral intervention used in the 2013 AHA/ACC/TOS guideline and subsequent systematic reviews.

Moderate intensity: Assign Intensity = Moderate if the intervention includes 6 to 13 sessions or 180 to 419 minutes (3 to 7 hours) of total contact time. Typical characteristics include monthly contact, 1 to 2 BCT modules, and regular feedback mechanism.

Low intensity: Assign Intensity = Low if the intervention includes fewer than 6 sessions or less than 180 minutes (less than 3 hours) of total contact time. Typical characteristics include baseline plus 1 to 2 follow-ups, education only, and no active BCT.

3.4 Multicomponent lifestyle intervention intensity

Primary approach: composite assessment based on the intensity levels of individual components.

3.4.1 Classification criteria

High intensity: Assign Intensity = High if at least two components are classified as High intensity, or one component is classified as High intensity and at least one component is classified as Moderate intensity.

Moderate intensity: Assign Intensity = Moderate if one component is classified as High intensity and all other components are classified as Low intensity, or at least two components are classified as Moderate intensity.

Low intensity: Assign Intensity = Low if all components are classified as Low intensity, or only one component is classified as Moderate intensity and all other components are classified as Low intensity.

3.5 Pharmacological dose and regimen coding

Pharmacological intervention arms will be coded according to the individual agent or fixed-dose combination, dose, dosing frequency, route of administration, titration schedule, treatment duration, and concomitant lifestyle intervention. Dose-based or combination nodes will be examined only when supported by sufficient independent trials and network connectivity is preserved.

**References**

1.	Bull FC, Al-Ansari SS, Biddle S, et al. World health organization 2020 guidelines on physical activity and sedentary behaviour. *Br J Sports Med*. 2020;54(24):1451-1462. doi:10.1136/bjsports-2020-102955

2.	Jensen MD, Ryan DH, Apovian CM, et al. 2013 AHA/ACC/TOS guideline for the management of overweight and obesity in adults. *Circulation*. 2014;129(25_suppl_2):S102-S138. doi:10.1161/01.cir.0000437739.71477.ee

3.	US Preventive Services Task Force. Behavioral weight loss interventions to prevent obesity-related morbidity and mortality in adults: US preventive services task force recommendation statement. *JAMA*. 2018;320(11):1163-1171. doi:10.1001/jama.2018.13022

4.	Jayedi A, Soltani S, Emadi A, Zargar MS, Najafi A. Aerobic exercise and weight loss in adults: A systematic review and dose-response meta-analysis. *JAMA Netw Open*. 2024;7(12):e2452185. doi:10.1001/jamanetworkopen.2024.52185
