# Supplementary material for: Systematic review and network meta-analysis of interventions for adults with simple obesity: Study protocol
Source: PLoS One. 2026 Oct 8;21(10):e0351589. doi: 10.1371/journal.pone.0351589 (PMC13648986; doi:10.1371/journal.pone.0351589)
Supplement: Supplementary file 4 [file pone.0351589.s004.docx]

**S4 Appendix. Data conversion and variance derivation**

All numerical data will be converted to a common analytic format before synthesis. For continuous outcomes, the preferred format will be the arm-level change-from-baseline mean and standard deviation. For binary outcomes, the preferred format will be arm-level event counts and total participants. When the preferred format is unavailable, effect estimates and variances will be derived using prespecified rules based on the reported summary statistics. The data-conversion hierarchy will be as follows: directly reported change mean and SD; change mean with SE or 95% CI; baseline and endpoint mean and SD; median with IQR or range; and, for binary outcomes, reported RR or OR with 95% CI when raw event counts cannot be recovered.

**4.1 Conversion of reported uncertainty measures to standard deviations**

When an arm-level mean is reported with a standard error, the standard deviation will be derived as:

$$SD=SE\times\sqrt{n},$$

where n is the corresponding arm-specific sample size.

When an arm-level mean is reported with a 95% confidence interval, the standard deviation will be derived as:

$$SD=\sqrt{n}\times\frac{Upper-Lower}{3.92}.$$

For small samples, the normal critical value will be replaced by the corresponding t-distribution critical value when sufficient information is available. When medians and interquartile ranges are reported, the standard deviation will be approximated as:

$$SD\approx\frac{Q3-Q1}{1.35}.$$

When range data are used, the standard deviation will be approximated as Range/4 for n < 70 and Range/6 for n ≥ 70, unless a more precise sample-size-based method is applicable.

Median values will be treated as approximate means only when the distribution is judged to be approximately symmetric. Potentially skewed data will be flagged when the relationship between the median and quartiles suggests marked asymmetry or when the mean-to-SD relationship is implausible for a non-negative outcome. Such data will be retained in the main dataset only when no better source is available and will be considered in sensitivity analyses where appropriate.

**4.2 Derivation of change-from-baseline values**

For continuous outcomes, change-from-baseline values will be prioritized because they better reflect within-arm treatment response over follow-up. When only baseline and endpoint means are available, the mean change will be derived as:

$$Change mean=End mean-Baseline mean.$$

The standard deviation of the change score will be derived as:

$$SD_{change}=\sqrt{SD_{baseline}^{2}+SD_{end}^{2}-2r\times SD_{baseline}\times SD_{end}},$$

where r is the within-participant correlation between baseline and endpoint values. When a trial reports the baseline SD, the endpoint SD, and the change SD, the trial-specific correlation will be recovered algebraically as:

$$r=\frac{SD_{baseline}^{2}+SD_{end}^{2}-SD_{change}^{2}}{2\times SD_{baseline}\times SD_{end}}$$

Recovered correlations will be pooled across trials by outcome, and the median outcome-specific value will be used as the default for trials in which the correlation cannot be recovered. Values outside the interval 0 to 1 will be treated as implausible and excluded from this calculation. If fewer than five trials permit recovery for a given outcome, r = 0.5 will be used as a conservative default. Because body weight and central-obesity measures are highly stable within participants, the recovered correlations are expected to be substantially higher than 0.5, and the influence of this assumption will be examined in sensitivity analyses using r = 0.5, the recovered median, and r = 0.9.

**4.3 Derivation of percentage body weight change**

When percentage body weight change is directly reported, the reported change mean and SD will be used.

When absolute change from baseline is reported as a mean with an SD and the baseline mean is available, percentage body weight change will be derived as:

$$Mean_{\%change}\approx\frac{Mean_{change}}{\mu_{B}}\times100$$

and its standard deviation as:

$$SD_{\%change}\approx\frac{100\times SD_{change}}{\mu_{B}}$$

This approximation treats the arm-level baseline mean as a fixed scaling constant and therefore does not propagate between-participant variability in baseline weight. Because the coefficient of variation of baseline weight is small relative to the variability of weight change, the resulting understatement of the variance is expected to be minor. This route will take precedence over the endpoint-based derivation below, because it uses the reported change SD directly and requires no assumption about the baseline–endpoint correlation. When only baseline and endpoint body weight are available, percentage body weight change will be approximated as:

$$Mean_{\%change}\approx\frac{End mean-Baseline mean}{Baseline mean}\times100.$$

When the SD of percentage change is not reported, it will be derived using a delta-method approximation:

$$SD_{\%change}\approx100\times\sqrt{\frac{SD_{end}^{2}}{\mu_{B}^{2}}+\frac{\mu_{E}^{2}SD_{baseline}^{2}}{\mu_{B}^{4}}-\frac{2\mu_{E}rSD_{baseline}SD_{end}}{\mu_{B}^{3}}},$$

where $\mu_{B}$ is the baseline mean, $\mu_{E}$ is the endpoint mean, $SD_{baseline}$ is the baseline SD, $SD_{end}$ is the endpoint SD, and r is the baseline–endpoint correlation. The correlation will be recovered or imputed as described in Section 4.2. Derived percentage-change values will be flagged in the dataset to distinguish them from directly reported percentage-change outcomes.

**4.4 Unit harmonization**

Outcome means and SDs will be standardized to the analysis units used: body weight in kilograms, percentage body-weight change in percentage points, waist circumference in centimetres, and ratio outcomes on their reported scales. Health-related quality of life will be retained on the scale reported by each trial, because these outcomes will be pooled as standardized mean differences. For instruments on which lower scores denote better quality of life, the direction of effect will be reversed by multiplying the mean by −1, leaving the SD unchanged, so that all trials share a common orientation. The instrument, subscale, and score range used by each trial will be recorded.

**4.5 Binary outcomes and effect estimates**

For binary outcomes, including at least 5% weight loss, withdrawal due to adverse events, and gastrointestinal adverse events, raw arm-level event counts and total participants will be extracted wherever possible. If only an odds ratio or risk ratio with a 95% confidence interval is reported and raw event counts cannot be reconstructed, the standard error of the log effect estimate will be derived as:

$$SE_{\ln\left( OR \right)}=\frac{\ln\left( Upper \right)-\ln\left( Lower \right)}{3.92},$$

with the same formula applied to $\ln\left( RR \right)$when a risk ratio is reported. These estimates will be entered into generic inverse-variance analyses where appropriate.

If an odds ratio needs to be approximated as a risk ratio for descriptive interpretation and the comparator-arm event risk is available, the following approximation will be used:

$$RR\approx\frac{OR}{1-P_{c}+P_{c}\times OR},$$

where $P_{c}$will represent the event probability in the comparator arm. Such conversions will be used cautiously and will not be preferred over raw event data.

**4.6 Sparse binary outcomes and continuity correction**

For studies in which at least one trial arm, but not all trial arms, reports zero events for a binary outcome, a continuity correction of 0.5 will be added to all cells for that study before the risk ratio is calculated. Studies reporting zero events in all trial arms will not contribute to the relative-effect synthesis because they will provide no information on the risk ratio, but they will be retained in the study characteristics and descriptive safety summaries. The numbers of studies requiring continuity correction and studies excluded from the relative-effect synthesis because of zero events in all arms will be reported. Sensitivity analyses excluding studies requiring continuity correction will be conducted when sufficient studies are available.

**4.7 Cluster-randomized trials**

For cluster-randomized trials, effect estimates and variances adjusted for clustering will be used when reported by the original trial, such as estimates obtained from mixed-effects models, generalized estimating equations, or other cluster-adjusted analyses. If a cluster-randomized trial reports only unadjusted arm-level data, the design effect will be calculated as:

$$DE=1+\left( m-1 \right)\times ICC,$$

where $m$will represent the average cluster size and $ICC$will represent the intracluster correlation coefficient. The adjusted standard error will then be calculated as:

$$SE_{\text{adjusted}}=SE_{\text{unadjusted}}\times\sqrt{DE}.$$

Alternatively, the effective sample size will be calculated as $n/DE$when this approach is more appropriate for the effect measure and available data structure.

**4.8 Multi-arm trials**

For network meta-analysis models that retain correlations among effect estimates from multi-arm trials, all eligible arms will be entered without artificially splitting the shared comparator. For pairwise descriptive analyses or other calculations requiring independent comparisons, shared comparator groups will be handled in a manner that avoids double counting. When necessary, the comparator sample size and number of events will be split proportionally across comparisons.

When multiple similar intervention arms need to be combined, the combined mean will be calculated as:

$$M_{\text{combined}}=\frac{n_{1}M_{1}+n_{2}M_{2}}{n_{1}+n_{2}},$$

and the combined standard deviation will be calculated as:

$$SD_{\text{combined}}=\sqrt{\frac{\left( n_{1}-1 \right)SD_{1}^{2}+\left( n_{2}-1 \right)SD_{2}^{2}+\frac{n_{1}n_{2}\left( M_{1}-M_{2} \right)^{2}}{n_{1}+n_{2}}}{n_{1}+n_{2}-1}}.$$

**4.9 Documentation of derived values**

All converted or derived values will be flagged in the analytic dataset. For each derived value, the source statistic, conversion formula, sample size used, correlation assumption where applicable, and derivation category will be recorded. The derivation category will indicate whether the value was directly reported, converted from a reported uncertainty measure, derived from baseline and endpoint data, or reconstructed from an effect estimate.

Values requiring unverifiable assumptions will not be used in the primary analysis unless no alternative data are available. In such cases, their influence will be assessed through sensitivity analyses where feasible.
