# Supplementary material for: Systematic review and network meta-analysis of interventions for adults with simple obesity: Study protocol
Source: PLoS One. 2026 Oct 8;21(10):e0351589. doi: 10.1371/journal.pone.0351589 (PMC13648986; doi:10.1371/journal.pone.0351589)
Supplement: Supplementary file 5 [file pone.0351589.s005.docx]

**S5 Appendix. Statistical methods for NMA**

Pairwise meta-analyses and network meta-analyses will be conducted in R using the **netmeta** package within a frequentist random-effects framework. Analyses will be performed separately for each outcome and each analysis scenario, and the versions of R and all analytical packages will be reported in the completed review. The primary reference treatment will be the non-active comparator.

For continuous outcomes, intervention effects will be summarized as mean differences when outcomes are measured on a common scale, including percentage body-weight change, absolute body-weight change, waist circumference, waist-to-hip ratio, and waist-to-height ratio. For health-related quality of life, which is assessed with different instruments across trials, intervention effects will be summarized as standardized mean differences using Hedges' g, and scores will be oriented so that higher values indicate better quality of life before pooling. Trials reporting quality of life with the same instrument will additionally be summarized as mean differences in a supplementary analysis to aid clinical interpretation. For binary outcomes, including at least 5% weight loss, withdrawal due to adverse events, and gastrointestinal adverse events, treatment effects will be summarized as risk ratios. Negative mean differences for weight and central-obesity outcomes will indicate greater benefit of the active intervention, whereas risk ratios greater than 1 will indicate a higher probability of the binary event.

Random-effects models will be used because clinical and methodological heterogeneity is expected across trials, including differences in baseline BMI, follow-up duration, intervention intensity, comparator content, and population classification. For each outcome-specific network, a common between-study heterogeneity variance will be assumed across comparisons and will be estimated using restricted maximum likelihood. Heterogeneity will be assessed using Cochran’s $Q$, the between-study variance $\tau^{2}$, and $I^{2}$. These statistics will be reported for pairwise comparisons where applicable and for each outcome-specific network.

Multi-arm trials will be handled within the network meta-analysis model while preserving the correlations induced by shared trial arms. For descriptive pairwise analyses, shared comparator groups will be split or similar arms will be combined only when necessary to avoid double counting, as described in S4 Appendix. Disconnected networks will not be forced into a single analysis. If an outcome network contains disconnected components, analyses will be restricted to connected subnetworks, and indirect comparisons across disconnected subnetworks will not be presented.

Treatment rankings will be summarized using P-scores within each outcome-specific frequentist NMA. Ranking results will be interpreted as secondary descriptive summaries rather than as stand-alone clinical recommendations. Clinical interpretation will prioritize effect estimates, confidence intervals, safety outcomes, certainty of evidence, and the consistency of findings across primary and sensitivity analyses.

The plausibility of the transitivity assumption will be assessed using both design-based and empirical considerations. At the design stage, transitivity will be supported by the prespecified treatment-node framework, in which pharmacological nodes are defined at the level of the individual agent or fixed-dose combination and different doses of the same agent and regimens combining that agent with lifestyle interventions are assigned to the same drug-level node in the primary analysis; by the requirement that active lifestyle interventions contain structured, protocol-driven components; and by the alignment of placebo, usual care, minimal advice, and no-intervention arms within the non-active comparator node. Dose-level and combination-level subdivision will be undertaken only in additional analyses when supported by sufficient independent trials and when network connectivity is preserved.

Empirically, potential effect modifiers will be summarized by trial, treatment node, and direct comparison. These will include baseline BMI, baseline weight or central-obesity measures where available, age, proportion of female participants, prevalence of major cardiometabolic comorbidities, follow-up duration, intervention intensity, comparator type, and risk of bias. Their distributions will be examined using summary tables, box plots, and appropriate descriptive or statistical comparisons to determine whether the transitivity assumption is plausible.

Inconsistency, or incoherence between direct and indirect evidence, will be assessed using local and global approaches where the network structure permits. Locally, node-splitting methods will be used to compare direct and indirect evidence for the same treatment contrast. Globally, design-by-treatment interaction models will be used to evaluate inconsistency across the network. These analyses will be interpreted alongside the amount of direct evidence, the presence of closed loops, heterogeneity, and clinical differences between trials.

Small-study effects and potential publication bias will be assessed using graphical and statistical methods when sufficient evidence is available. For pairwise comparisons with at least 10 trials, conventional funnel plots will be examined. At the network level, comparison-adjusted funnel plots will be used to examine small-study patterns across treatment contrasts. Egger regression tests or Peters tests will be applied when the number of studies is sufficient and heterogeneity is not extreme. Funnel-plot asymmetry will not be interpreted as definitive evidence of publication bias without considering clinical heterogeneity, event rates, trial size, risk of bias, and selective outcome reporting. This approach will follow the protocol-specified plan for publication-bias assessment.

Sensitivity analyses will be conducted to examine the robustness of the primary findings. These will include the all-population analysis, analyses including shorter-follow-up trials, exclusion of studies at high risk of bias, Bayesian re-estimation under plausible prior distributions, and other scenario-specific analyses where applicable. Bayesian sensitivity analyses, inclusion of shorter-follow-up trials, and exclusion of high-risk-of-bias studies will constitute the key robustness checks.

Benefit–risk visualization will be used as a descriptive tool to jointly display weight-loss benefit and tolerability risk. For interventions with both effectiveness estimates and estimates of withdrawal due to adverse events, mean differences in body-weight change will be plotted against risk ratios for withdrawal due to adverse events. These plots will not constitute formal multicriteria decision analyses and will not generate composite benefit–risk scores. Their purpose will be to support clinical interpretation by showing whether larger weight-loss effects are accompanied by a higher risk of adverse-event-related discontinuation. Interventions without estimable values for both dimensions will not be displayed in the benefit–risk plot.
