# Supplementary material for: Systematic review and network meta-analysis of interventions for adults with simple obesity: Study protocol
Source: PLoS One. 2026 Oct 8;21(10):e0351589. doi: 10.1371/journal.pone.0351589 (PMC13648986; doi:10.1371/journal.pone.0351589)
Supplement: Supplementary file 6 [file pone.0351589.s006.docx]

**S6 Checklist. PRISMA-P checklist**

| **Section and topic** | **Item No** | **Checklist item** |
| --- | --- | --- |
| **ADMINISTRATIVE INFORMATION** | | |
| Title: |  |  |
| Identification | 1a | Title: “Systematic review and network meta-analysis of interventions for adults with simple obesity: study protocol” |
| Update | 1b | Not applicable |
| Registration | 2 | PROSPERO registration number: CRD420251236477 |
| Authors: |  |  |
| Contact | 3a | Corresponding author: Lei Tian, Centre for Pharmacoeconomics and Outcomes Research, China Pharmaceutical University, Nanjing, 211198, China. Email: cputianlei@163.com |
| Contributions | 3b | Lingyao Sun and Lizhen Xu conceived the review question, designed the study, and drafted the first version of the protocol. Dachuang Zhou and Wentao Ye developed and refined the eligibility criteria, search strategies, and data extraction forms. Jun Wang and Xuan Yin provided clinical expertise on obesity management and contributed to the interpretation of the planned analyses. Lei Tian critically reviewed all sections of the protocol, provided methodological guidance, and supervised the overall project. All authors revised the manuscript for important intellectual content, approved the final version of the protocol, and agreed to be accountable for all aspects of the work. |
| Amendments | 4 | Reported in the Study status and timeline section. Any amendments made after PROSPERO registration will be transparently documented. |
| Support: |  |  |
| Sources | 5a | This research received no specific grant from any funding agency in the public, commercial, or not-for-profit sectors. |
| Sponsor | 5b | None |
| Role of sponsor or funder | 5c | None |
| **INTRODUCTION** | | |
| Rationale | 6 | Reported in the Introduction. Existing systematic reviews generally focus on individual intervention categories or mixed disease-specific populations. This review addresses the evidence gap for broadly recruited adults in whom obesity, rather than a specific chronic disease, is the principal clinical condition. |
| Objectives | 7 | Reported in the Abstract, Introduction, and Methods.  • Participants: Adults aged ≥18 years with simple obesity, defined using regional obesity criteria and the prespecified comorbidity classification framework.  • Interventions: Individual pharmacological agents or fixed-dose combinations, and dietary, physical activity, behavioural, and multicomponent lifestyle interventions.  • Comparators: Active intervention nodes and non-active comparators. Placebo and no active intervention will be combined in the primary analysis and separated in sensitivity analyses when network connectivity permits.  • Outcomes: The principal primary outcome is percentage change in body weight. Key secondary outcomes include achievement of ≥5% weight loss, absolute body-weight change, separate measures of central obesity, health-related quality of life, and safety outcomes. |
| **METHODS** | | |
| Eligibility criteria | 8 | Reported in the Methods—Eligibility criteria, Interventions, Comparators, and Search strategy sections.  • Study design: Parallel-group individually randomized controlled trials, including cluster-randomized trials. Quasi-randomized trials, cross-over trials, non-randomized studies, observational studies, animal experiments, and in-vitro studies will be excluded.  • Participants: Adults aged ≥18 years who meet the diagnostic criteria for obesity applicable in the country or region where the trial was conducted. Obesity attributable to a clearly identified genetic syndrome or endocrine disorder and trials recruiting a specific chronic-disease population will be excluded or classified according to the prespecified comorbidity framework.  • Interventions and comparators: Eligible pharmacological treatments and structured dietary, physical activity, behavioural, or multicomponent lifestyle interventions, with active and non-active comparators classified according to the prespecified node definitions.  • Language: No language restrictions will be applied.. |
| Information sources | 9 | • Electronic databases: PubMed, Embase via Ovid, and the Cochrane Library.  • Trial registries: ClinicalTrials.gov and the International Clinical Trials Registry Platform Search Portal.  • Other grey literature: Doctoral and master’s theses and conference abstracts.  • Additional sources: Reference lists of included studies and relevant systematic reviews and network meta-analyses. |
| Search strategy | 10 | Reported in the Search strategy section and Appendix 1. Controlled vocabulary and free-text terms will be combined for population or condition, intervention, and randomized-trial design concepts. Searches will cover database inception to the final search date, with no language restrictions. Complete database-specific strategies are provided in Appendix 1. |
| Study records: |  |  |
| Data management | 11a | Reported in the Data extraction section. Data will be extracted independently by two reviewers using an electronic spreadsheet based on a standardized data extraction form. The form will be pilot-tested on at least five eligible randomized controlled trials and refined before formal use. |
| Selection process | 11b | Reported in the Study selection section. Two reviewers will independently screen titles and abstracts and subsequently assess full-text reports against prespecified eligibility criteria. Disagreements will be resolved by discussion or adjudication by a third reviewer. |
| Data collection process | 11c | Reported in the Study selection, Data extraction, and Appendix 4 sections. Two reviewers will independently extract data using a standardized and piloted form. Study authors will be contacted for missing information; when numerical data remain unavailable, prespecified data-conversion and variance-derivation methods will be applied where the reported statistics permit. |
| Data items | 12 | Reported in the Population, Interventions, Comparators, Outcomes, and Data extraction sections. Extracted items will include study and design characteristics; regional obesity definitions, BMI eligibility thresholds, baseline BMI and central-obesity measures; age, sex distribution, and metabolic or comorbidity status; intervention type, drug dose or non-pharmacological intensity, route, frequency, duration, adherence, and implementation characteristics; comparator type and regimen; outcome definitions and reported time points; and numerical data required to calculate effect sizes or variances, including event counts, means, standard deviations, medians, interquartile ranges, and related statistics. |
| Outcomes and prioritization | 13 | Reported in the Outcomes section. Percentage change in body weight is the principal primary outcome. The proportion of participants achieving at least 5% weight loss is a key secondary efficacy outcome, and absolute change in body weight is an additional weight-related outcome. Waist circumference, waist-to-hip ratio, and waist-to-height ratio will be analysed separately as measures of central obesity. Health-related quality of life is a patient-reported secondary outcome. Other secondary outcomes include low-density lipoprotein cholesterol, systolic blood pressure, fasting plasma glucose, serum uric acid, nonalcoholic fatty liver disease indices, treatment discontinuation or withdrawal due to adverse events, gastrointestinal adverse events, weight regain, and body image scores. |
| Risk of bias in individual studies | 14 | Reported in the Risk of bias assessment section. Two reviewers will independently assess risk of bias at the study level using the Cochrane RoB 2 tool and will classify each domain as low risk, some concerns, or high risk. Disagreements will be resolved through discussion or adjudication by a third reviewer. For cluster-randomized trials, additional design-specific issues will be considered, including recruitment after allocation, loss or imbalance of clusters, whether clustering was appropriately accounted for, contamination, and cluster-level co-interventions. |
| Data synthesis | 15a | Quantitative synthesis will be conducted separately for each outcome when studies can be mapped to the prespecified intervention nodes and form a clinically interpretable connected evidence network. Disconnected components will be analysed separately, and indirect comparisons will not be made across disconnected subnetworks. |
|  | 15b | Reported in the Data synthesis and Statistical implementation sections and Appendices 4 and 5.  • Effect measures: Mean differences will be used for continuous outcomes measured on a common scale, standardized mean differences when different instruments measure the same construct, and risk ratios for binary outcomes.  • Statistical models: Pairwise and network meta-analyses will be conducted in R using the netmeta package under a frequentist random-effects framework. A common between-study heterogeneity variance will be assumed across treatment comparisons and estimated using restricted maximum likelihood.  • Multi-arm trials: Within-study correlations arising from shared trial arms will be preserved in the network model.  • Missing and converted data: Prespecified data-conversion and variance-derivation methods will be applied when directly reported statistics are unavailable, and derived values will be documented as described in Appendix 4.  • Sparse binary outcomes: A continuity correction of 0.5 will be added to all cells of studies with zero events in some but not all arms. Studies with zero events in all arms will be excluded from risk-ratio synthesis but retained in descriptive summaries.  • Heterogeneity, transitivity, and inconsistency: Heterogeneity will be assessed using Cochran’s Q, τ², and I². Potential effect modifiers will be compared across trials, treatment nodes, and direct comparisons. Local inconsistency will be examined using node splitting and global inconsistency using design-by-treatment interaction models.  • Treatment ranking: Rankings will be summarized using P-scores and interpreted alongside effect estimates, 95% confidence intervals, and GRADE certainty assessments rather than as stand-alone evidence of treatment superiority. |
|  | 15c | Reported in the Interventions, Comparators, Subgroup and meta-regression, and Sensitivity analyses sections.  • Potential effect modifiers: Baseline BMI, age, intervention duration or follow-up duration, disease or comorbidity status, proportion of female participants, baseline central-obesity measures, and intervention intensity.  • Sensitivity and additional analyses: Bayesian models for the principal primary outcome; inclusion of trials with intervention duration <24 weeks; exclusion of studies at high risk of bias; duration-stratified analyses at 24 to <52 weeks, 52 to <104 weeks, and ≥104 weeks when data and network connectivity permit; intervention duration as a continuous effect modifier; separation of placebo and no-active-intervention comparator nodes when feasible; and more granular pharmacological or non-pharmacological nodes when supported by sufficient independent trials and preserved network connectivity.  • Credibility assessment: Important subgroup findings will be assessed using the ICEMAN tool. |
|  | 15d | Reported in the Study selection and Data synthesis sections. Disconnected network components will be analysed separately, and any narrative juxtaposition across subnetworks will be interpreted cautiously. |
| Meta-bias(es) | 16 | Reported in the Assessment of publication bias section. Conventional funnel plots will be examined for pairwise comparisons containing at least 10 trials, and comparison-adjusted funnel plots will be used at the network level. Egger’s or Peters’ tests will be applied when the number of studies and outcome structure are appropriate. Funnel-plot asymmetry will be interpreted in conjunction with heterogeneity, study size, risk of bias, and selective reporting. |
| Confidence in cumulative evidence | 17 | Reported in the Certainty of evidence section. The certainty of evidence for the principal primary outcome and key secondary outcomes will be assessed using the GRADE framework and categorized as high, moderate, low, or very low. Certainty ratings will be incorporated into the interpretation of effect estimates and treatment rankings. |
